# Antihyperglycaemic and organic protective effects on pancreas, liver and kidney by polysaccharides from *Hericium erinaceus* SG-02 in streptozotocin-induced diabetic mice

**DOI:** 10.1038/s41598-017-11457-w

**Published:** 2017-09-07

**Authors:** Chen Zhang, Juan Li, Chunlong Hu, Jing Wang, Jianjun Zhang, Zhenzhen Ren, Xinling Song, Le Jia

**Affiliations:** 1 College of Life Science, Shandong Agricultural University, Taian, 271018 China; 2 Chinese Academy of Agricultural Sciences, Beijing, 100081 China; 3 College of Forestry, Shandong Agricultural University, Taian, 271018 China; 4 The Central Hospital of Taian, Taian, 271000 China

## Abstract

The present work was designed to investigate the antihyperglycaemic and protective effects of two *Hericium erinaceus* intracellular polysaccharide (HIPS) purified fractions (HIPS1 and HIPS2) from mycelia of *H. erinaceus* SG-02 on pancreas, liver and kidney in streptozotocin (STZ)-induced diabetic mice. The supplementation of HIPS1 and HIPS2 significantly decreased the blood glucose (GLU) levels; suppressed the abnormal elevations of alkaline phosphatase (ALP), alanine aminotransferase (ALT), aspartate aminotransferase (AST), urea nitrogen (BUN) and creatinine (CRE) levels in serum; improved the antioxidant enzymatic (superoxide dismutase (SOD), glutathione peroxidase (GSH-Px) and catalase (CAT)) activities; and attenuated the pathological damage to these organs. The HIPS1 showed superior effects in antihyperglycaemia and organic protection than HIPS2 possible owing to the abundant functional groups (-NH_2_, -COOH and S=O) in HIPS1, indicating that *H. erinaceus* SG-02 could be used as a functional food and natural drug for the prevention of diabetes and its complications.

## Introduction

Diabetes mellitus (DM), the most significant chronic disease, which is characterized by hyperglycaemia and is associated with disturbances in carbohydrate, protein and fat metabolism, has become a global public health issue due to its prevalence and high morbidity^1^. Clinically, DM can be classified into two types: type I (insulin-dependent DM) and type II (non-insulin-dependent DM). The former (type I) is always accompanied with complications such as vision loss, renal failure and nerve damage, while the latter (type II) is characterized by peripheral insulin resistance and impaired insulin secretion, leading to cardiovascular system diseases^2^. Many factors have potential effects that raise the risk of DM, including viral infections, autoimmune diseases, unnatural diets, environmental factors, and other variables^3–6^. Recently, increasing literature reports of both clinical and experimental studies have demonstrated that oxidative stress plays a vital role in the progression of DM and its complications^7–11^. Streptozotocin (STZ), depending on its biotoxicity on pancreatic β-cells, which play important roles in the homeostasis of blood glucose through insulin synthesis, has been commonly used as an agent for experimentally inducing DM^10–12^. The cellular mechanisms of β-cell destruction may be correlated with local reactive oxygen species (ROS) and nitric oxide (NO), which are induced by cytokine stimulation. In addition, the liver and kidneys, the detoxifying organs of the body, are affected by STZ-induced oxidative stress, which causes structural damage. Antioxidant agents can increase the expression of antioxidant enzymes and alleviate oxidative stress in both serum and organs, which can enhance the resistance to DM^13–16^. Therefore, the antihyperglycaemic effects of some antioxidants should be investigated.

Clinically, insulin injection and oral administration of hypoglycaemic agents are two traditional therapies for treating DM^17^. Among such treatments, the control of postprandial hyperglycaemia is critical in early therapy for DM^18^. One therapeutic approach to decrease postprandial hyperglycaemia is to retard the absorption of glucose by inhibiting carbohydrate-hydrolysing enzymes, such as α-amylase and α-glucosidase, in digestive organs^19^. The inhibition rate for carbohydrate-hydrolysing enzymes could be regarded as a preliminary indicator for the effectiveness of therapeutic agents. Recently, common oral hypoglycaemic agents, such as sulfonylureas and biguanides, have been blamed for their side effects during long-time use and yield little therapeutic efficacy against diabetic complications^17^. Hence, alternative medicines and natural therapies with non-toxic properties have gained further academic attention. As an alternative approach, mushrooms have been widely used in medical applications due to the abundant bioactive compounds they contain, which have potential anticarcinogenic, anti-inflammatory, hypolipidaemic, antidiabetic and hepatinica activities^20^. Interestingly, it has previously been demonstrated that polysaccharides from mushrooms, such as *Pleurotus djamor* ^7^ *, Phellinus baumii* ^21^ and *Agrocybe cylindracea* ^13^, have significant effects in the treatment of DM and its complications.

*Hericium erinaceus* (*H. erinaceus*), an edible fungus that inhabits mountainous areas, has been used in traditional folk medicine and medicinal cuisine in the northeast territories of Asia^22^. Previous pharmacological studies have confirmed that *H. erinaceus* has potential medicinal applications in the treatment and prevention of hyperglycaemia, chronic bronchitis, cancer, arteriosclerosis, hypertension, hypercholesterolemia and leucopenia^23^. In recent years, basic studies on the chemical identification of the active ingredients of *H. erinaceus* have progressed, and polysaccharides have been identified as the main effective components. Submerged cultures have become a main source of polysaccharides, having the advantage of shorter incubation times and higher production levels compared with common cultures^22, 24^. To the best of our knowledge, up to this point, there have been no reports regarding any protective effects of mycelia polysaccharides from *H. erinaceus* SG-02 on pancreas, livers and kidneys in STZ-induced diabetic mice.

In this article, two new intracellular polysaccharides were successfully isolated from mycelia of *H. erinaceus* SG-02. Their antihyperglycaemic and inhibitory effects on carbohydrate-hydrolysing enzymes and their protective effects on pancreas, livers and kidneys in STZ-induced diabetic mice were investigated. Moreover, their monosaccharide compositions and infrared spectra were also analysed.

## Results

### Purification of *H. erinaceus* intracellular polysaccharide (HIPS)

**Figure 1:**
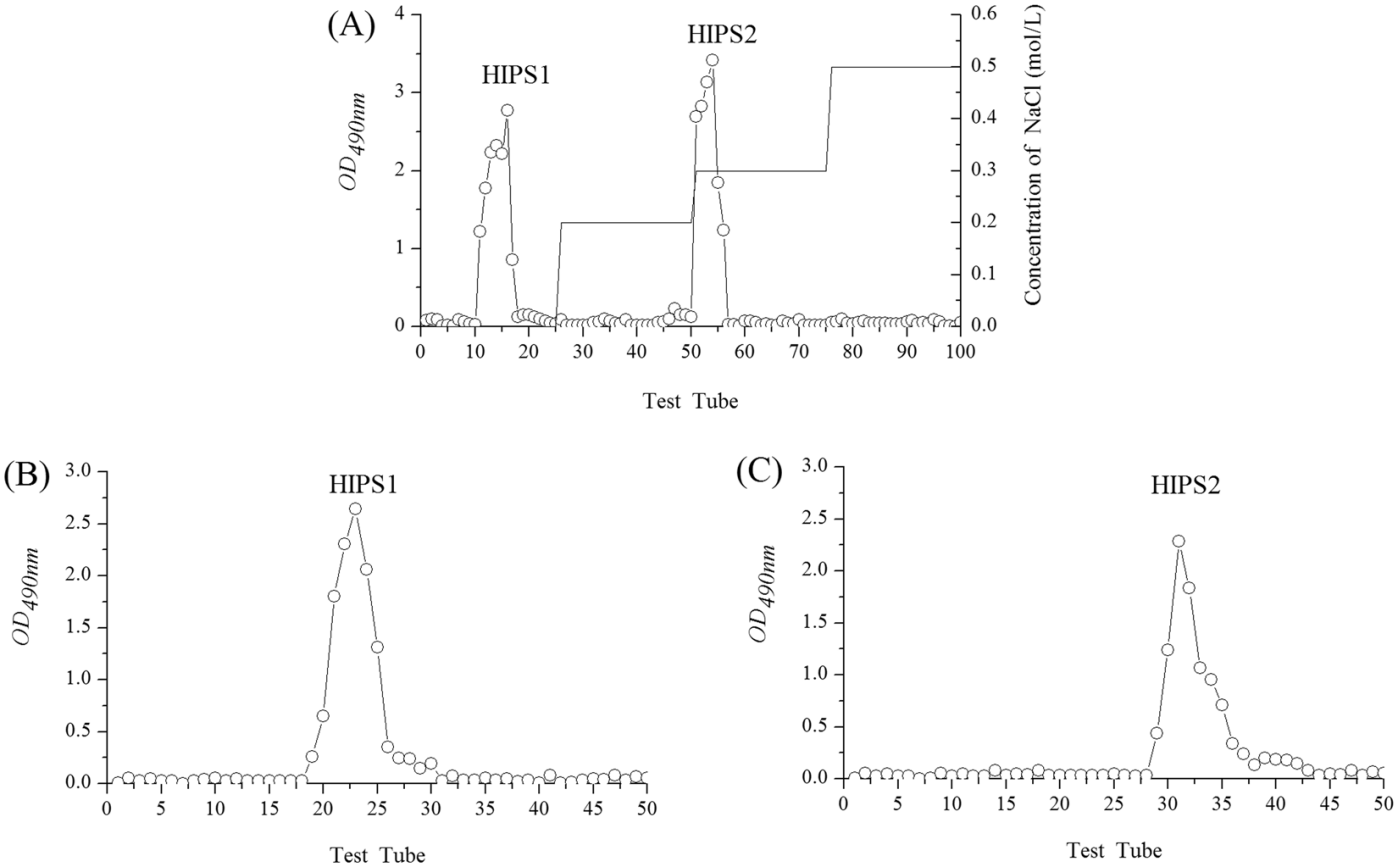
Elution profiles of HIPS1 and HIPS2. (**A**) Elution profiles of HIPS by DEAE-52 cellulose column chromatography with gradient of NaCl solution (0, 0.2, 0.3 and 0.5 mol/L), (**B**) Elution profiles of HIPS1, and (**C**) HIPS2 on Sephadex G-100 cellulose column.

As shown in Fig. 1, two elution peaks, HIPS1 and HIPS2, were purified via DEAE-52 anion-exchange chromatography. HIPS1 was a neutral polysaccharide, as it was eluted with distilled water, while HIPS2 was an acidic polysaccharide, as it was eluted with a 0.3 mol/L NaCl solution^25^. Individual peaks of both HIPS1 and HIPS2 were purified using a Sephadex G-100 cellulose column (Fig. 1B and C), demonstrating that the two fractions were both homogeneous and pure. The yields of HIPS1 and HIPS2 were 21.62 ± 0.74% and 32.75 ± 1.21%, respectively.

### Ultraviolet (UV) spectroscopy, Gas chromatography (GC) and Fourier transform infrared (FT-IR) analysis of polysaccharides

The UV scanning spectrums of HIPS1 and HIPS2 showed no absorption at 280 and 260 nm, indicating the polysaccharides were purified and free of nucleic acids and proteins.

**Figure 2:**
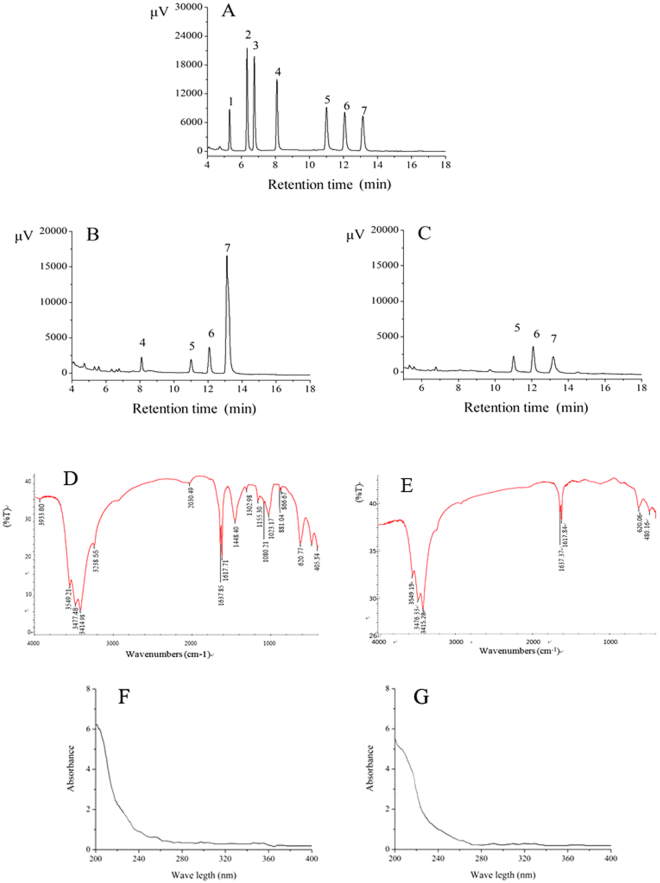
The gas chromatographs and FT-IR spectrums of polysaccharides. Gas chromatographs of (**A**) standard monosaccharides, (**B**) HIPS1 and (**C**) HIPS2; FT-IR spectrums of (**D**) HIPS1 and (**E**) HIPS2. Peaks: (1) Rha, (2) Rib, (3) Ara, (4) Xyl, (5) Man, (6) Gal and (7) Glc; UV scanning spectrums of (**F**) HIPS1 and (**G**) HIPS2.

The monosaccharide compositions of HIPS1 and HIPS2 are shown in Fig. 2. HIPS1 was composed of xylose (Xyl) (3.02%), mannose (Man) (4.36%), galactose (Gal) (9.41%) and glucose (Glc) (83.21%), with molar ratios of 1:1.2:2.6:23.1 (Fig. 2B), while HIPS2 contained Man, Gal and Glc, with mass percentages of 23.40%, 50.34% and 26.27%, and molar ratios of 1:2.15:1.12 (Fig. 2C), respectively.

FT-IR spectra of HIPS1 and HIPS2 are shown in Fig. 2. It is obvious that both HIPS1 and HIPS2 exhibited typical major -OH broad stretching bands for carbohydrates at approximately 3500–3400 cm^−1^. The absorption peaks at 1637.85 and 1637.37 cm^−1^ are attributed to the stretching vibration of C=O. The bands at approximately 1618 cm^−1^ indicate the presence of -NH_2_ in the two samples. The absorption peak for HIPS1 at 2030.49 cm^−1^ is a characteristic absorption peak for C≡C^26^, and the bands at approximately 1450 cm^−1^ indicate the presence of -COOH groups in HIPS1^27^. The band at approximately 1153 cm^−1^ suggests valent vibrations of the C-O-C bond and a glycosidic bridge in HIPS1^23^, while α-glucan^28^ had an additional band at 1026 cm^−1^. The characteristic absorption peak at 866.03 cm^−1^ indicates the presence of a furan ring in HIPS1^13^, while the additional weak peak at approximately 620.83 cm^−1^ describes S=O vibrations in HIPS1^26, 29^.

### Inhibitory effects on α-amylase and α-glucosidase activities

**Figure 3:**
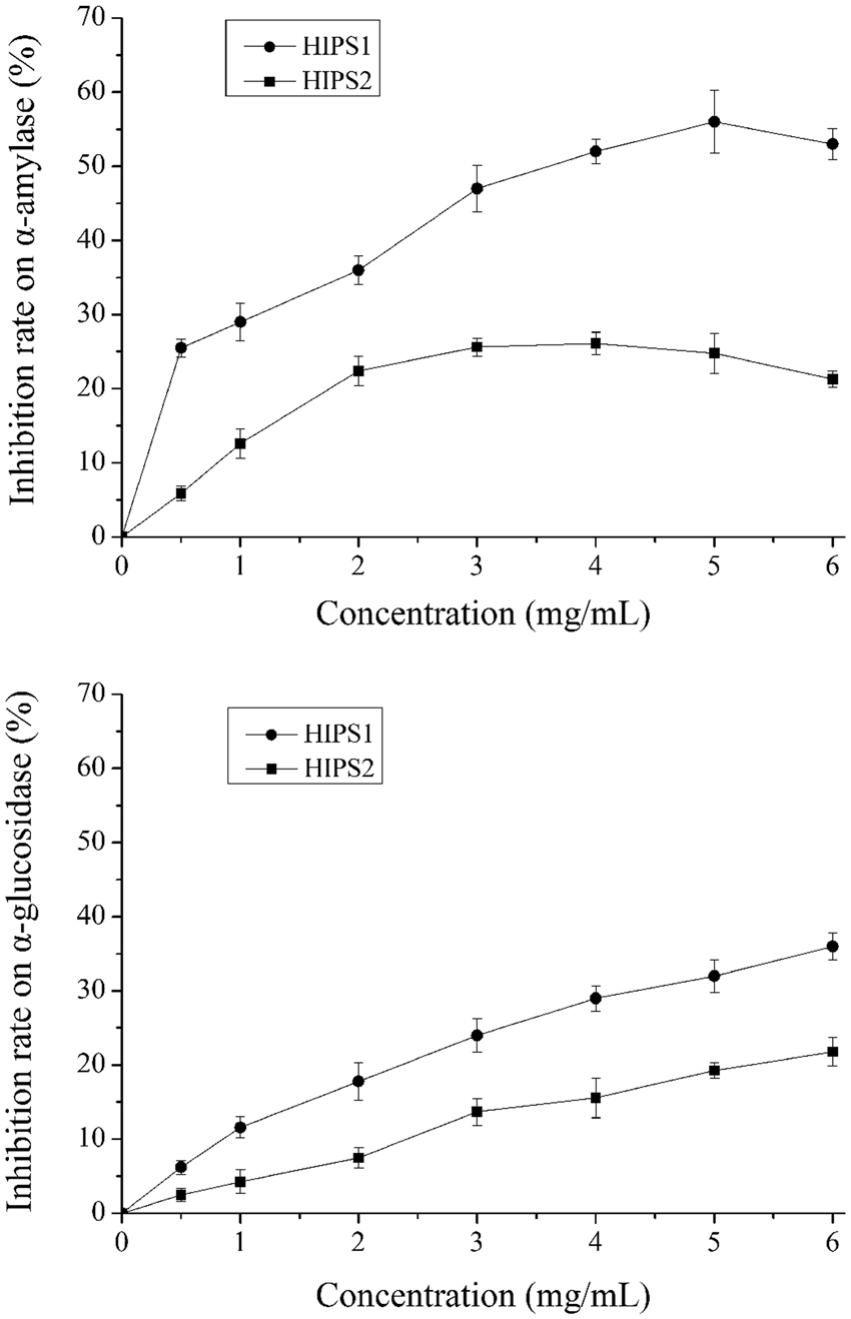
The inhibition rates of HIPS1 and HIPS2 on α-amylase and α-glucosidase. The values are reported as the means ± SD (n = 3).

The inhibitory effects of HIPS1 and HIPS2 on α-amylase and α-glucosidase activities were investigated in the present work. As shown in Fig. 3, the inhibitory activities of HIPS2 on α-amylase and α-glucosidase were lower than those of HIPS1. The α-amylase inhibition rate of HIPS1 (53.27 ± 2.16%) was maximal at a concentration of 5 mg/mL, which was 32.09% higher than that for HIPS2. The 50% inhibitory concentration (IC50) value for HIPS1 on α-amylase activity was 3.42 ± 0.29 mg/mL, and the inhibition rate declined at concentrations greater than 5 mg/mL. At a concentration of 6 mg/mL, the α-glucosidase inhibition rate of HIPS1 was 36.64 ± 1.83%, which was 14.21% higher than that for HIPS2.

### Effects of HIPS1 and HIPS2 on blood glucose (GLU) levels, body weights, and pancreas, liver and kidney indices

The results for GLU levels, body weight, and pancreas, liver and kidney indices in STZ-induced diabetic mice are listed in Table 1. Before treatment, the GLU levels of the diabetic mice were markedly increased than those of normal mice (Table 1, *P* < 0.05). After treatment, the GLU levels in the six dose groups were significant decreased than that in the MC groups, indicating that the pathological increases could be suppressed by HIPS1 and HIPS2 administration at different doses (*P* < 0.05). Specifically, compared with the MC group, the final GLU levels in the H-HIPS1, M-HIPS1, L-HIPS1, H-HIPS2, M-HIPS2 and L-HIPS2 groups were reduced by 51.65 ± 9.11, 38.77 ± 5.48, 34.21 ± 6.54, 37.05 ± 6.80, 32.56 ± 9.67 and 28.93 ± 5.94%, respectively. Additionally, both HIPS1 and HIPS2 showed effects in reducing GLU levels but the values remained different in comparison with that in NC groups among the test doses.

**Table 1:** Effects of HIPS1 and HIPS2 on the GLU levels, body weights, as well as index of pancreas, liver and kidney.

Group	GLU levels (mmol/L)		Body weight (g)		Pancreas index (g/100 g)	Liver index (g/100 g)	Kidney index (g/100 g)
	Pre-treatment	Post-treatment	Pre-treatment	Post-treatment			
NC	4.13 ± 0.21a	4.18 ± 0.12a	25.23 ± 1.21a	34.29 ± 1.12a^#^	1.76 ± 0.13a	15.41 ± 0.89a	4.27 ± 0.19a
MC	13.82 ± 0.15c	15.14 ± 0.56b^#^	24.16 ± 0.95a	25.24 ± 0.97d	2.78 ± 0.27d	23.78 ± 1.76d	9.64 ± 0.71d
PC	14.76 ± 0.17b	5.35 ± 0.41f^#^	23.93 ± 0.32a	32.77 ± 1.13ab^#^	2.31 ± 0.25c	18.16 ± 0.79a	5.82 ± 0.48ab
H-HIPS1	14.05 ± 0.58c	7.32 ± 0.82e^#^	24.89 ± 0.68a	31.38 ± 1.42ab^#^	1.89 ± 0.21ab	18.38 ± 1.06a	5.87 ± 1.03ab
M-HIPS1	13.67 ± 0.32c	9.27 ± 0.17d^#^	25.45 ± 0.86a	30.19 ± 1.17abc^#^	1.92 ± 0.16ab	19.43 ± 1.73ab	6.81 ± 0.92b
L-HIPS1	13.87 ± 0.13c	9.96 ± 0.45cd^#^	24.97 ± 0.79a	28.96 ± 0.59bc^#^	2.23 ± 0.11bc	21.51 ± 0.53bc	7.21 ± 1.27bc
H-HIPS2	13.98 ± 0.49c	9.53 ± 0.47d^#^	25.41 ± 0.47a	29.05 ± 1.51bc^#^	2.21 ± 0.23bc	20.96 ± 0.67ab	8.21 ± 1.52c
M-HIPS2	14.87 ± 0.25b	10.21 ± 0.91cd^#^	25.47 ± 1.18a	29.21 ± 0.92c^#^	2.26 ± 0.22bc	22.15 ± 0.82c	8.54 ± 0.96c
L-HIPS2	13.99 ± 0.31c	10.76 ± 0.34c^#^	25.03 ± 1.31a	27.56 ± 1.21b^#^	2.58 ± 0.15cd	23.21 ± 2.23cd	9.21 ± 1.19cd

The values are reported as the means ± SD (n = 10 for each group). Means with the same letter are not significantly different (*P* < 0.05). ^#^Significant difference compared to Pre-treatment (*P* < 0.05).

The body weights of pre-treatment and post-treatment diabetic mice were also determined. At the onset of the experiments, there was no significant difference (*P* < 0.05) between the body weights of normal and diabetic mice, while the diabetic mice without any treatment had emaciated bodies compared with the seven treatment groups at the end of the study. Specifically, the body weights of mice in the NC groups were significantly (*P* < 0.05) increased during the treatment. However, the diabetic mice in the MC group exhibited significant weight loss compared with the NC group (Table 1, *P* < 0.05). When compared with that in the MC groups, the mice in H-HIPS1, M-HIPS1, L-HIPS1, H-HIPS2 and M-HIPS2 groups showed significant improvements on the body weights (*P* < 0.05). And the results also indicated that HIPS1 showed stronger effects in improving the body weights, which was reflected by the paralleled values in comparison with that in the NC groups.

Furthermore, the indices for the pancreas, liver and kidneys in the mice were also investigated, and the results are listed in Table 1. Significant increases in the pancreas, liver and kidney indices were observed in the diabetic mice (MC) group compared with those in the NC group (*P* < 0.05). However, the three indices were decreased after treatment with HIPS1 and a high dose of HIPS2. Especially in the H-HIPS1 group, the pancreas, liver and kidney indices of the mice were reduced by 32.01 ± 2.16, 22.71 ± 2.94 and 39.11 ± 3.32%, respectively, compared with those in the MC group. However, HIPS2 was much less effective than HIPS1 in restoring the organ indexes, especially at the low dose.

### Effects of HIPS1 and HIPS2 on serum biochemistry

The activities and levels of albumin (ALB), alkaline phosphatase (ALP), alanine aminotransferase (ALT), aspartate aminotransferase (AST), urea nitrogen (BUN) and creatinine (CRE) in serum are clinically used as biochemical markers for early liver and kidney damage. As shown in Table 2, mice exhibited liver and kidney damage after injection with STZ, as evidenced by significant increases in the serum levels of ALP, ALT, AST, BUN and CRE (*P* < 0.05) and decreased ALB levels. Dramatically, when compared with that in the MC groups, the activities of ALT, AST and ALP, as well as the levels of BUN and CRE were significantly (*P* < 0.05) decreased, while the ALB levels were increased by treatment with HIPS1 at the three tested dosages. However, only the high dose of HIPS2 (600 mg/kg) was effective in maintaining the above effectiveness. Particularly, the serum biochemical parameters for mice in the H-HIPS1 groups were similar to that in normal mice, indicating the high dose of HIPS1 was much more effective than the other doses of HIPS1 and HIPS2. In the present work, HIPS1 could suppress the abnormal elevations of ALP, ALT, AST, BUN and CRE levels and the decline in ALB levels in diabetic mice, indicating that the polysaccharides extracted from *H. erinaceus* SG-02 had potential protective effects against STZ-induced liver and kidney damage. Additionally, supplementation with glibenclamide could achieve effects similar to supplementation with HIPS1 (Table 2).

**Table 2:** Effects of HIPS1 and HIPS2 on serum biochemistry in different groups.

Group	ALB (g/L)	ALP (U/L)	ALT (U/L)	AST (U/L)	BUN (mmol/L)	CRE (μmol/L)
NC	35.65 ± 2.47a	121.67 ± 13.66a	37.41 ± 3.56a	116.42 ± 13.36a	6.23 ± 0.19a	60.27 ± 2.19a
MC	12.74 ± 3.78f	183.13 ± 20.14d	126.38 ± 15.37e	217.83 ± 30.55f	8.17 ± 0.21d	93.53 ± 10.38d
PC	30.79 ± 3.80bc	138.21 ± 9.76ab	47.93 ± 7.31ab	142.29 ± 17.45bc	6.57 ± 0.44ab	66.22 ± 2.95ab
H-HIPS1	31.21 ± 3.52b	139.52 ± 10.59b	44.98 ± 5.99a	135.27 ± 16.11ab	6.81 ± 0.33ab	66.32 ± 4.31ab
M-HIPS1	29.28 ± 4.3bc	150.67 ± 8.38bc	62.13 ± 10.96b	151.12 ± 11.65c	7.21 ± 0.11bc	75.22 ± 3.34b
L-HIPS1	23.78 ± 5.62de	159.53 ± 13.55bc	88.74 ± 16.51c	188.67 ± 9.64e	7.86 ± 0.31cd	78.66 ± 4.92c
H-HIPS2	25.95 ± 1.49cd	161.07 ± 15.33bcd	83.77 ± 16.59c	180.29 ± 15.49d	7.95 ± 0.12cd	75.49 ± 7.47c
M-HIPS2	20.87 ± 3.38e	168.42 ± 11.57cd	109.21 ± 18.65d	186.44 ± 13.87de	8.09 ± 0. 28cd	86.08 ± 6.85cd
L-HIPS2	17.99 ± 3.05e	172.69 ± 15.94cd	115.39 ± 20.19de	194.41 ± 22.51e	8.26 ± 0.39cd	87.24 ± 3.77d

The values are reported as the means ± SD (n = 10 for each group). Means with the same letter are not significantly different (*P* < 0.05).

### Effects of HIPS1 and HIPS2 on GSH peroxide (GSH-Px), superoxide dismutase (SOD), catalase (CAT), and malondialdehyde (MDA)

**Figure 4:**
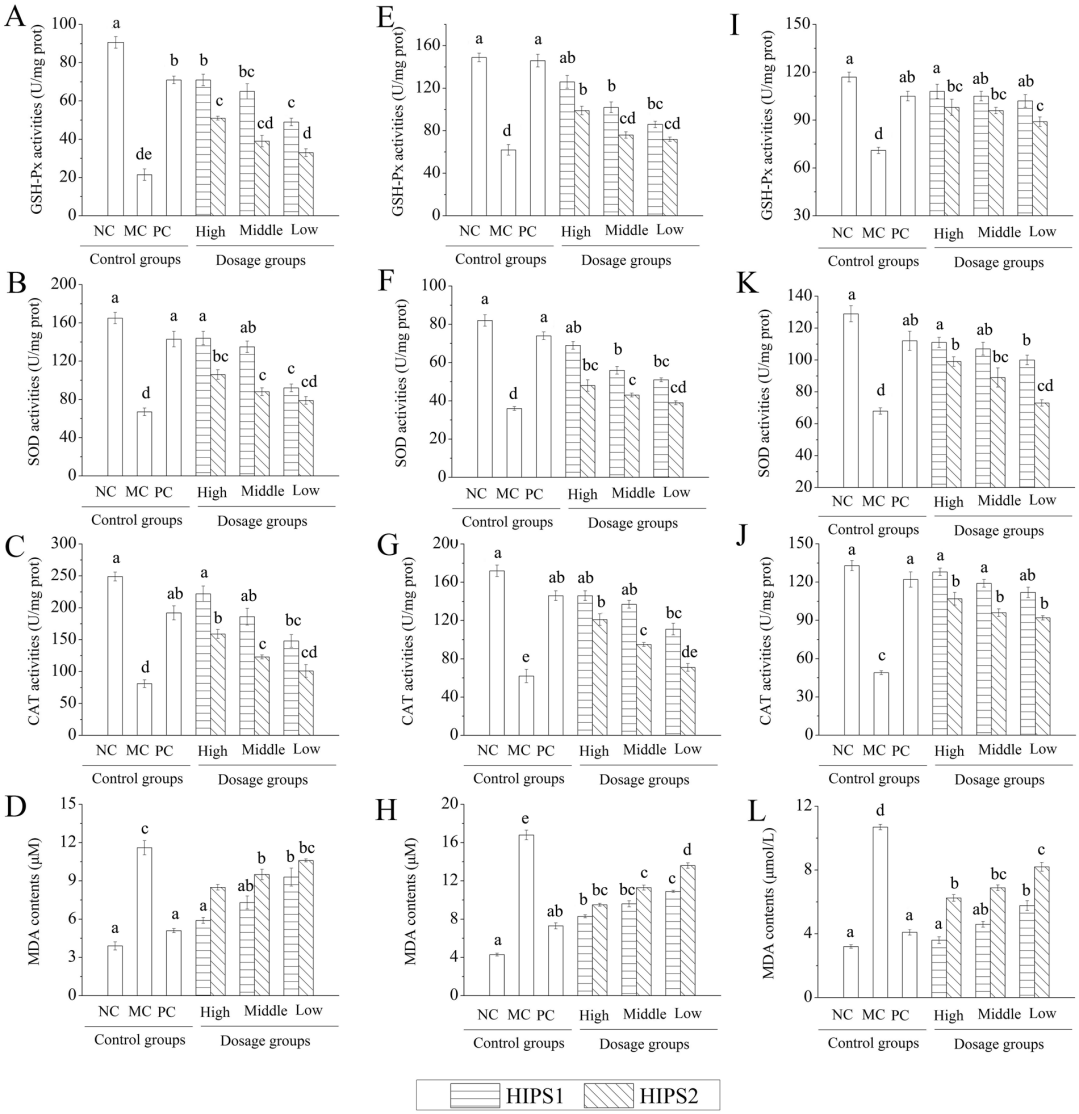
Effects of HIPS1 and HIPS2 on GSH-Px, SOD, CAT, and MDA in liver (**A**–**D**), kidney (**E**–**H**) and pancreas (**I**–**L**). The values are reported as the means ± SD (n = 10 for each group). Means with the same letter are not significantly different (*P* < 0.05).

To study the effects of HIPS1 and HIPS2 administration on enzyme activities and free radical production, GSH-Px, SOD and CAT activities and MDA levels were measured in the three organs (Fig. 4). At the end of the experiment, the GSH-Px, SOD and CAT activities in STZ-induced diabetic mice (MC groups) were significantly reduced (*P* < 0.05), while MDA levels were markedly increased (*P* < 0.05) compared with those in the NC groups, indicating that the pancreas, liver and kidney all suffered oxidative stress. As shown in Fig. 4, the abnormal physiological and biochemical changes in the three organs of diabetic mice were restored by HIPS1 and HIPS2 administration. The GSH-Px, SOD and CAT activities in the liver reached maximum values of 72.75 ± 4.38, 158.68 ± 16.42 and 223.14 ± 26.12 U/mg protein, respectively, in the H-HIPS1 group, values that were significantly higher than those in the MC group (20.98 ± 3.26, 65.85 ± 5.31 and 74.96 ± 9.41 U/mg protein), respectively, (*P* < 0.05 for all), and with no significant difference compared to the NC group (Fig. 4A–C). The hepatic MDA levels reached 5.98 ± 0.23 μmol/L in the H-HIPS1 group, which was reduced by 49.14 ± 6.72% compared with those in the MC groups (Fig. 4D).

Similar results were also observed for GSH-Px, SOD and CAT activities in the kidney (Fig. 4E–G) and pancreas (Fig. 4I–K). When compared with the MC group, significant (*P* < 0.05) increases were observed after the administration of HIPS1 (including the three doses) and HIPS2 (including the high dose and middle dose). The MDA levels for the kidney (Fig. 4H) and pancreas (Fig. 4L) also declined after treatment with the polysaccharides. Glibenclamide-treated mice also manifested significant increases in SOD, GSH-Px, and CAT activities, and decreases in the MDA levels in the three organs compared with diabetic mice (*P* < 0.05).

The relevant indices in the H-HIPS1 group were approximately equal to those in the NC group. This conclusion demonstrated that the high dose of HIPS1 exhibited strong potential antioxidant effects against oxidative stress in the liver, kidney and pancreas.

### Histopathological observations of the pancreas, liver and kidney

**Figure 5:**
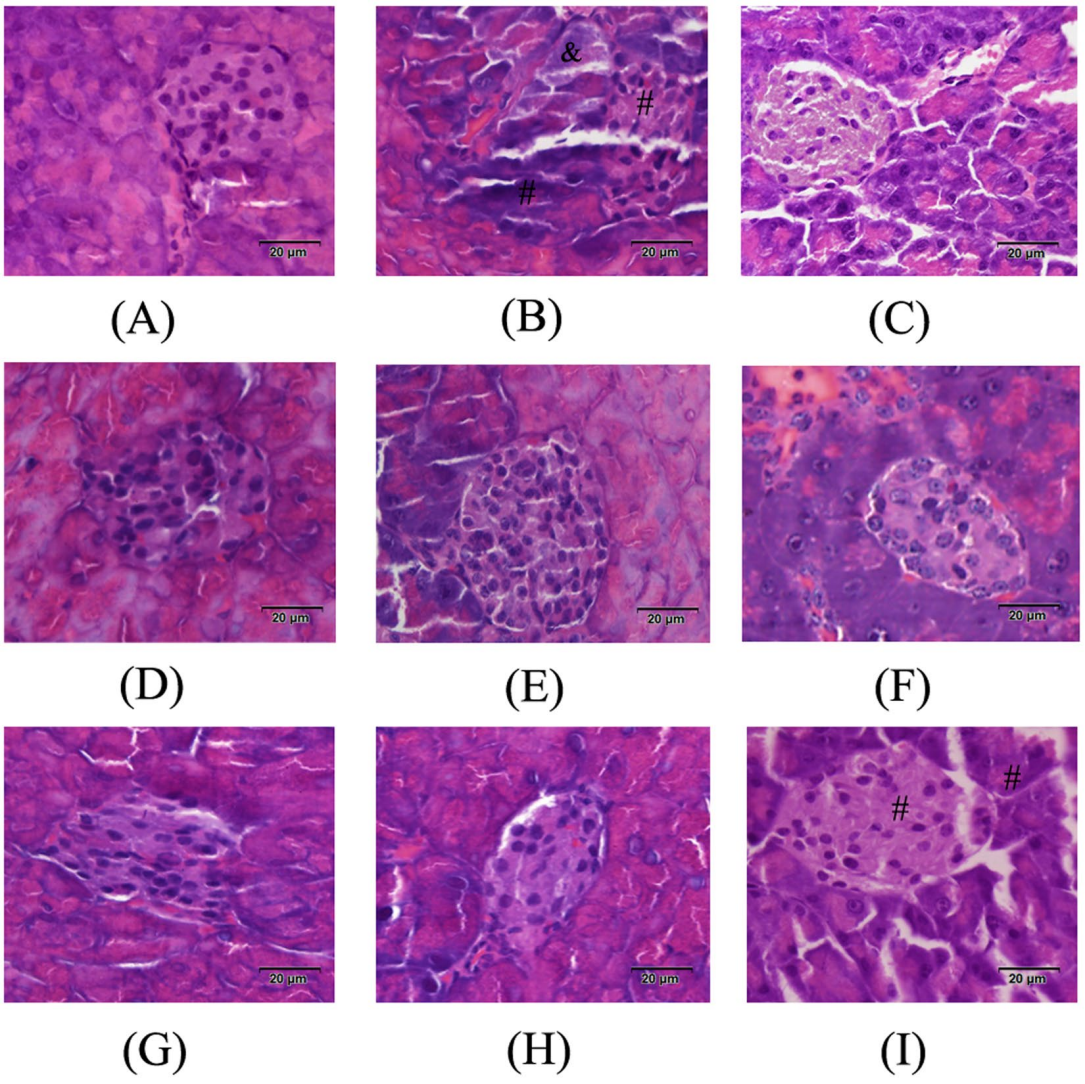
Effects of HIPS1 and HIPS2 on pancreatic histopathology in STZ-induced diabetic mice (hematoxylin-eosin staining, 400×). (**A**) NC, (**B**) MC, (**C**) PC, (**D**) H-HIPS1, (**E**) M-HIPS1, (**F**) L-HIPS1, (**G**) H-HIPS2, (**H**) M-HIPS2 and (**I**) L-HIPS2 & showed the infiltration of lymphocytes, ^#^showed the hypochromatosis and the disappearance of cell borders.

**Figure 6:**
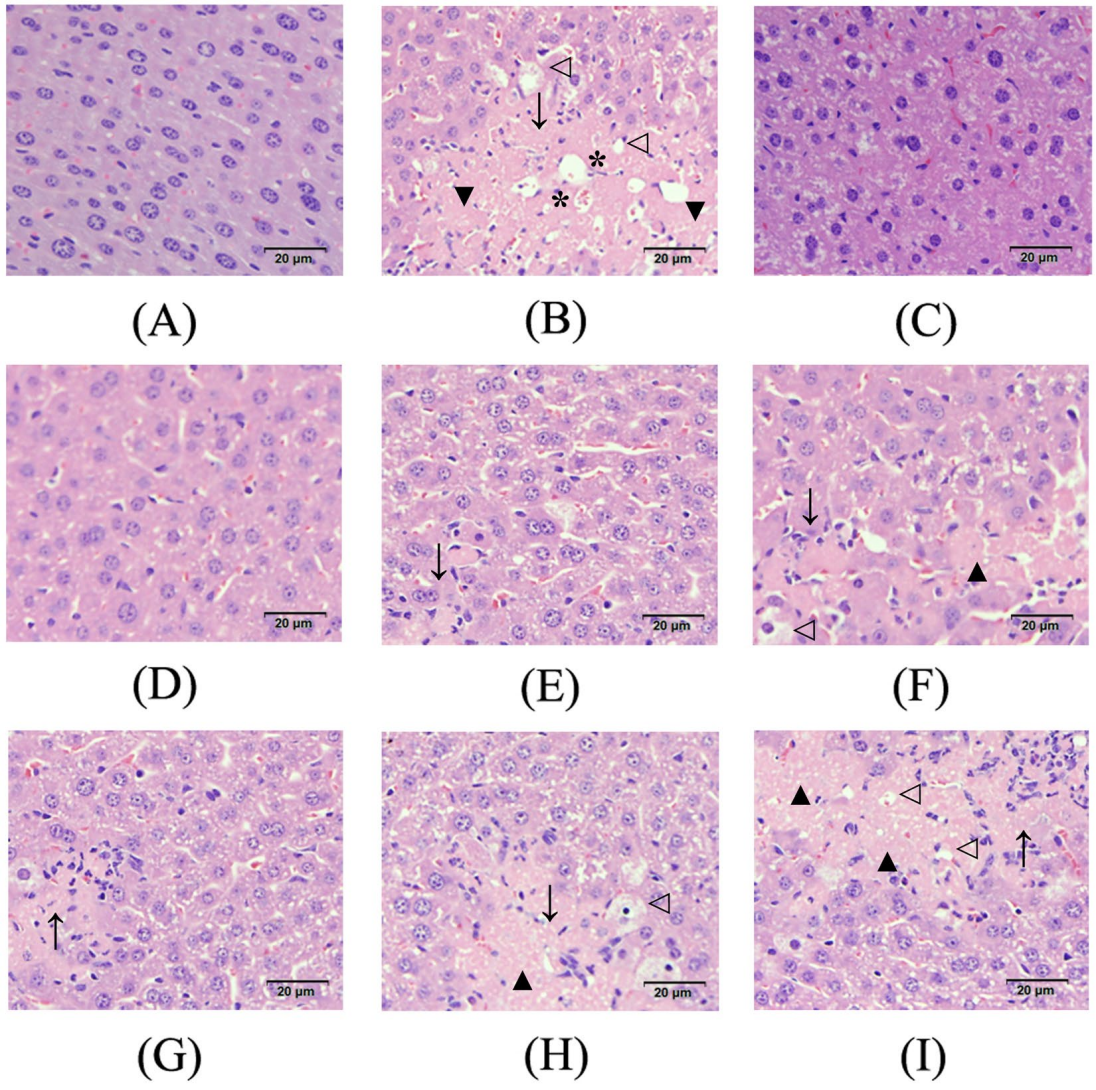
Effects of HIPS1 and HIPS2 on hepatic histopathology in STZ-induced diabetic mice (hematoxylin-eosin staining, 400×). (**A**) NC, (**B**) MC, (**C**) PC, (**D**) H-HIPS1, (**E**) M-HIPS1, (**F**) L-HIPS1, (**G**) H-HIPS2, (**H**) M-HIPS2 and (**I**) L-HIPS2. Arrows showed necrotic zones, triangles indicated hypochromatosis, hollow triangle showed fatty degeneration and *showed the perivascular inflammatory cell infiltration.

**Figure 7:**
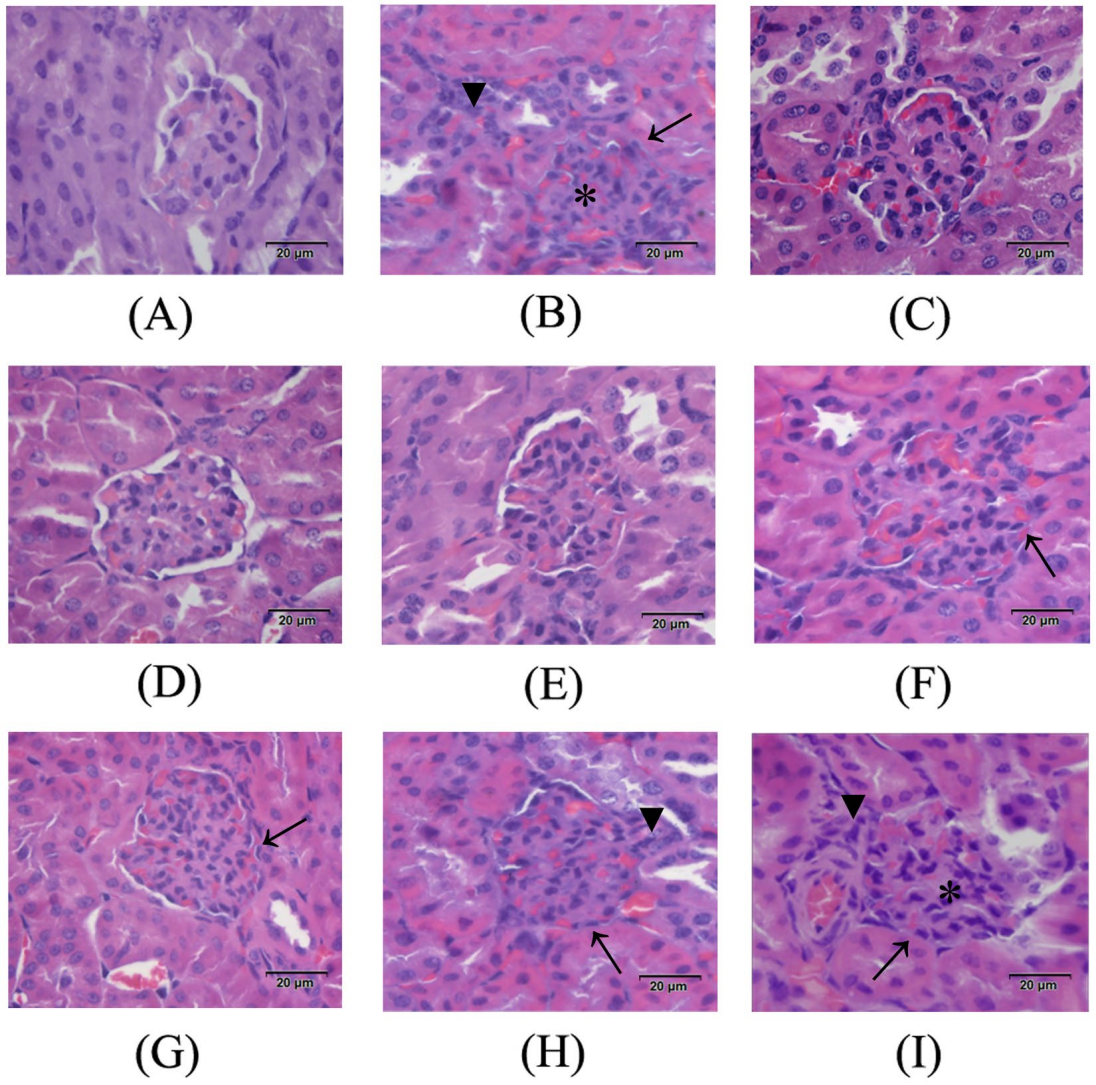
Effects of HIPS1 and HIPS2 on kidney cortex histopathology in STZ-induced diabetic mice (hematoxylin-eosin staining, 400×). (**A**) NC, (**B**) MC, (**C**) PC, (**D**) H-HIPS1, (**E**) M-HIPS1, (**F**) L-HIPS1, (**G**) H-HIPS2, (**H**) M-HIPS2 and (**I**) L-HIPS2. Arrows showed glomerular augmentation and triangles showed mesangial matrix proliferation, while *showed the loss of interstitial space.

Histopathological observations of the pancreas, liver and kidney were performed using haematoxylin and eosin (HE) staining (Figs 5–7).

The effects of HIPS1 and HIPS2 on pancreatic tissues are shown in Fig. 5. Severe degeneration in the pancreas appeared in the STZ-induced diabetic mice, including lymphocyte infiltration, hypochromatosis and the disappearance of cell borders in the pancreatic islets (Fig. 5B). After treatment with HIPS1 at all doses and HIPS2 at the high and middle doses, improvement in the pancreatic tissues could be observed, which could be evidenced by decreased infiltration and more integrated cell structure in the pancreatic islets compared with the mice in the MC group (Fig. 5D–I). These results indicated that treatment with polysaccharides from *H. erinaceus* SG-02 could repair islet damage and improve the structural integrity of pancreatic islet beta-cells and tissues.

As shown in Fig. 6A, the hepatocyte morphology was normal, and the hepatic cells were arranged in a regular manner with abundant cytoplasm, distinct cell borders and normal central nuclei. In the MC group, liver damage was observed in the mice treated with STZ. The changes in hepatocyte morphology were characterized by perivascular inflammatory cell infiltration, fatty degeneration, hypochromatosis and the disappearance of cell borders (Fig. 6B). In the dosage groups, treatment with HIPS1 (600 and 400 mg/kg) and HIPS2 (600 mg/kg) showed effects in ameliorating the liver damage (Fig. 6D,E and G). Especially for mice in the H-HIPS1 groups, the liver structures were similar to normal liver architectures (Fig. 6D).

The changes in kidney cortex histology are shown in Fig. 67. Compared with the mice in the NC groups with normal architecture (Fig. 7A), glomerular augmentation, glomerular swelling, mesangial matrix proliferation and loss of interstitial space were observed in mice in the MC group after STZ injection (Fig. 7B), indicating that kidney damage occurred in the diabetic mice. After the treatments with HIPS1 at high and middle doses, these pathological changes were attenuated, which were evidenced by the normal morphology of glomerulus in Fig. 7D and C.

Interestingly, the histopathological analysis of the pancreas and liver was consistent with the results of enzyme activities and MDA levels as biochemical indicators of their function (Table 2 and Fig. 4). Histological studies of the kidney cortices of mice in the H-HIPS1 and PC groups demonstrated similarities to those in the NC group, which echoed the results of the biochemical assays (Table 2 and Fig. 4).

### Acute toxicity

In the acute toxicity assays, the mice treated with HIPS1 and HIPS2 did not exhibit any gross behavioural changes (including irritation, restlessness, respiratory distress, abnormal locomotion and catalepsy) or toxic symptoms (such as decreased food intake or shaggy hair) either immediately or during the post-treatment period compared with the control group, indicating that these polysaccharides were essentially non-toxic substances.

## Discussion

Increasing data indicate enhanced oxidative stress and changes in antioxidant capacity in both clinical and experimental forms of DM that are key mechanisms in the pathogenesis of diabetic complications^30^. STZ injection has been used to establish an experimental hyperglycaemia model, owing to its high toxicity in stimulating the generation of H_2_O_2_ in β-cells, which may induce lipid peroxidation and the depletion of antioxidant enzymes, and even worse, organic damage^31–33^. Previous publications have reported decreases in antioxidant enzyme activities, including GSH-Px, SOD and CAT, in the pancreas, liver and kidney of diabetic mice, in agreement with our present conclusions^34^. Interestingly, the reductions in enzyme activities and the increase of MDA levels were markedly suppressed after treatment with HIPS1 or HIPS2 (Fig. 4), demonstrating that the polysaccharides may inhibit oxidative damage to pancreatic, hepatic and nephritic tissue. Pancreas, one of the most vital organs of polysaccharide metabolism, maintains homeostasis of blood glucose through insulin synthesis. STZ exhibits toxicity on pancreatic β-cells, leading to pancreatic damage, and results in disordered insulin secretion^32, 33^. As sensitive organs, the liver and kidneys have a great capacity to detoxify toxic substances, and they play pivotal roles in glucose metabolism *in vivo* ^31^. Therefore, the pathological damages that are inflicted on the liver and kidneys by hepatotoxic and nephrotoxic agents appear to be fatal in diabetes^35, 36^. Furthermore, in diabetic animals, the oxidative stress that is induced by free radicals can affect the functions of the pancreas, liver and kidneys^37^. In the present work, as shown in Figs 5–7, the STZ-induced diabetic mice exhibited serious damage to the pancreas, liver and kidneys, including cell necrosis, hypochromatosis, fatty degeneration and glomerular proliferation. Interestingly, this damage was considerably recovered by HIPS1 and HIPS2 administration, indicating that the damage to the pancreas, liver and kidneys in STZ-induced diabetic mice can be protected and repaired by intervention with HIPS1 and HIPS2.

High blood glucose levels are the main characteristic of DM^1^. In STZ-induced diabetic mice, high GLU levels and increased uptake of food and water indicate the successful establishment of the model^38^. One therapeutic approach for decreasing hyperglycaemia is to retard the absorption of glucose by inhibition of carbohydrate hydrolysis enzymes, such as α-amylase and α-glucosidase. Hence, it is important to investigate the inhibition of α-amylase and α-glucosidase to analyse the antihyperglycaemic effects^19, 39^. In the present study, HIPS1 and HIPS2 produced inhibitory effects against the two enzymes at different concentrations, and HIPS1 was more effective than HIPS2, apparently due to the different structures of the polysaccharides. Nevertheless, Chiba noted that the inhibitory effects may be associated with the individual structures of the enzymes^40^.

Blood has direct contact with most of the tissues in the animal body, and pathological changes are likely to be reflected in levels of some proteins and enzymes in blood samples^21^. Enhanced ALB levels and decreased activities of ALP, ALT and AST in serum have been used clinically as biochemical markers for liver damage^41, 42^. BUN and CRE, endogenous by-products that are released into body fluids and excreted by glomerular filtration, have been widely used in the clinic to reflect the physical status of the kidneys^43^. In the present study, changes in the activities of ALB, ALP, ALT, and AST and in serum levels of BUN and CRE were suppressed by HIPS1 and HIPS2 administration, indicating the stabilization of plasma membranes and repair of STZ-induced pancreatic, hepatic and nephritic damages. These results are also consistent with our previous studies^8^.

The present findings suggest that HIPS1 and HIPS2 from *H. erinaceus* SG-02 are novel bioactive compounds with potential anti-diabetic and organic protection effects. Moreover, HIPS1 was much more efficient than HIPS2 in preventing the diabetes-induced organs damages, with the possible mechanism might be due to the monosaccharide composition and bond types. Figure 2 shows that HIPS1 contains Xly and a high percentage of Glc in the monosaccharide composition compared with HIPS2. Furthermore, HIPS1 contains more active radicals and functional groups, such as -NH_2_, -COOH and S=O, than HIPS2. These structural characteristics might contribute to the scavenging capacities of ROS. Moreover, HIPS1 and HIPS2, especially HIPS1, could indirectly relieve oxidative damage to the pancreas, liver and kidneys by improving the antioxidant enzyme activities and reducing MDA levels (Fig. 4), possibly resulting in the amelioration of tissue necrosis and inflammatory damage in these organs. Otherwise, the repair capacities of HIPS1 and HIPS2 on the organs may involve a reduction of insulin secretion or stimulation of insulin release^44^ and may indirectly participate in inflammatory pathways^45^, possibilities that require further study.

## Methods

### Ethics statement

All experiments were performed in accordance with the guidelines and regulations of the ethics committee of the Shandong Agricultural University and the Animals (Scientific Procedures) Act of 1986 (amended 2013).

All experimental protocols were submitted to and approved by the ethics committee of the Shandong Agricultural University in accordance with the Animals (Scientific Procedures) Act of 1986 (amended 2013).

### Materials

The *H. erinaceus* SG-02 strain used in this experiment was preserved in the Fungi and Application Laboratory of Shandong Agriculture University (Taian, China) and was maintained on a potato dextrose agar (PDA) slant. The diagnostic kits for analysing SOD, GSH-Px, and CAT activities and MDA levels were purchased from the Nanjing Jiancheng Bioengineering Institute (Nanjing, China). The standard monosaccharide samples, including rhamnose (Rha), ribose (Rib), arabinose (Ara), Xyl, Glc, Man and Gal, were provided by the Merck Company (Darmstadt, Germany) and the Sigma Chemical Company (St. Louis, MO, USA). All other reagents and chemicals used in the present work were analytical reagent grade and were supplied by local chemical suppliers.

### Isolation and purification of polysaccharides

*H. erinaceus* SG-02 was initially maintained on PDA slants and then inoculated into 500 mL of liquid medium containing glucose (10 g), peptone (1.5 g), yeast extract, (2 g), KH_2_PO_4_ (1 g) and MgSO_4_ (0.5 g). After 25 days of fermentation, the mycelia of *H. erinaceus* SG-02 were collected and cleaned three times with distilled water. The dry mycelia were shattered and extracted with distilled water at 80 °C for 3 h and centrifuged at 3000 rpm for 10 min, after which the precipitate was discarded. The supernatant was mixed with anhydrous ethanol (4 °C overnight) to obtain the crude polysaccharide, which was deproteinized using Sevag reagent (chloroform/*n*-butanol, 5:1, v/v) and labelled as HIPS.

HIPS (1 g) was dissolved in 15 mL of distilled water and purified on a DEAE-cellulose column (26 × 400 mm) at a flow rate of 2.0 mL/min and eluted with sodium chloride solutions at concentrations of 0, 0.2, 0.3 and 0.5 mol/L. The major eluate was then collected separately and purified by gel permeation chromatography (Sephadex G-100 column AQ3, 1.3 × 50 cm). The above steps were repeated twenty times, and the main fractions were lyophilized by vacuum freeze-drying (Labconco, USA) for further study.

### UV spectroscopy, GC and FT-IR analysis of polysaccharides

The UV scanning spectrum was determined by microplate spectrophotometer (Dynex Spectra MR, Dynex Technologies, USA)

The monosaccharide composition was determined by GC (GC-2010, Shimadzu, Japan) according to the method of Sheng, *et al*.^46^. The monosaccharide compositions were identified by comparing the retention times with the standards, and the monosaccharide contents were calculated using area normalization methods.

Infrared spectra of the samples were recorded using an infrared spectrometer (Nicolet 6700, Thermo Fisher Scientific, USA) with a range of 4000–400 cm^−1^ ^47^.

### Inhibition assay on α-amylase and α-glucosidase activities

The HIPS fractions were stored at −80 °C prior to the assay of α-amylase inhibition. The reaction mixture, including 0.2 mL of α-amylase (6 U/mL) and 0.2 mL of polysaccharide solution (1.0 to 6.0 mg/mL, dissolved in 0.2 mol/L phosphate buffer, pH 6.6), was activated by adding the starch substrate solution (0.4 mL, 1%, w/w) and was processed at 37 °C for 10 min, followed by termination with 2 mL of DNS reagent (1% 3,5-dinitrosalicylic acid and 12% sodium potassium tartrate in 0.4 mol/L sodium hydroxide)^48^. The sample was maintained in boiling water for 10 min and then diluted with 15 mL of distilled water in an ice bath. The α-amylase activity was determined by measuring the absorbance at 540 nm. The IC50 value was defined as the concentration of polysaccharide that produced an inhibition rate of 50% under the assay conditions^19^.

1 $${\rm{Inhibition}}\,{\rm{rate}}\,( \% )=(1-({{\rm{A}}}_{{\rm{i}}}-{{\rm{A}}}_{{\rm{j}}})/{A}_{c})\times 100$$

where A_c_ was the absorbance of the mixture that includes 0.2 mL of phosphate buffer, 0.4 mL of starch solution and 0.2 mL of α-amylase solution; A_i_ was the absorbance of the mixture that includes 0.2 mL of sample, 0.4 mL of starch solution and 0.2 mL of α-amylase solution; and A_j_ was the absorbance of the mixture that includes 0.4 mL of phosphate buffer, 0.2 mL of α-amylase solution and 0.2 mL of sample.

Inhibition of α-glucosidase activity was measured according to reported methods with some modifications^19^. The α-glucosidase (0.2 mL, 3.75 U/mL) was premixed with the samples (dissolved in 0.2 mol/L phosphate buffer, pH 6.8) at various concentrations (0.5 to 6.0 mg/mL), and 6 mmol/L p-nitrophenyl-α-D-glucopyranoside (in phosphate buffer) as a substrate was added to the mixture to initiate the reaction. The reaction was incubated at 37 °C for 30 min and stopped by adding 6 mL of 0.1 mol/L sodium carbonate. The α-glucosidase activity was determined by measuring the absorbance at 400 nm.

2 $${\rm{Inhibition}}\,{\rm{rate}}\,( \% )=(1-({{\rm{A}}}_{{\rm{i}}}-{{\rm{A}}}_{{\rm{j}}}){/{\rm{A}}}_{{\rm{c}}})\times 100$$

where A_c_ was the absorbance of the mixture that includes 0.2 mL of phosphate buffer, 0.2 mL of p-nitrophenyl-α-D-glucopyranoside solution and 0.2 mL of α-glucosidase solution; A_i_ was the absorbance of the mixture that includes 0.2 mL of sample, 0.2 mL of p-nitrophenyl-α-D-glucopyranoside solution and 0.2 mL of α-glucosidase solution; and A_j_ was the absorbance of the mixture that includes 0.2 mL of phosphate buffer, α-glucosidase solution and 0.2 mL of sample.

### Animal experiments

Ninety Kunming strain mice (4 weeks old, weight: 20 ± 2 g, purchased from Taibang Biological Products Ltd. Co. (Taian, China)) were acclimated for one week under the following conditions: temperature (23 ± 1 °C), humidity (55 ± 5%) and a 12-h light-dark cycle, during which time they had free access to food and water *ad libitum*. After the acclimation, the mice were induced to become diabetic by an intraperitoneal injection with 120 mg/kg STZ (freshly dissolved in sodium citrate buffer, pH 4.5), while the NC group was injected intraperitoneally with citrate buffer^49^. The GLU levels in the blood samples obtained from the tail of each mouse were measured 48 h after the last STZ injection using an ACCU-CHEK blood glucose metre (Roche, Basel, Switzerland). Mice with GLU levels greater than 13.3 mmol/L were considered successful models of diabetes^50^ and were randomly allocated to the MC group, the PC group and six dosage groups (including L-HIPS1, L-HIPS2, M-HIPS1, M-HIPS2, H-HIPS1 and H-HIPS2), which contained ten mice per group. Different groups were administered different solutions intragastrically as follows:

NC group (n = 10): normal control. MC group (n = 10): diabetic control + distilled water. PC group (n = 10): diabetic control + glibenclamide (10 mg/kg). H-HIPS1 group (n = 10): diabetic control + HIPS1 (600 mg/kg). M-HIPS1 group (n = 10): diabetic control + HIPS1 (400 mg/kg). L-HIPS1 group (n = 10): diabetic control + HIPS1 (200 mg/kg). H-HIPS2 group (n = 10): diabetic control + HIPS2 (600 mg/kg). M-HIPS2 group (n = 10): diabetic control + HIPS2 (400 mg/kg). L-HIPS2 group (n = 10): diabetic control + HIPS2 (200 mg/kg).

The treatments lasted for two weeks. At the end of the experiment, all mice were weighed and sacrificed using a diethyl ether anaesthetic treatment after fasting overnight^51^.

The organic index for the pancreas, liver and kidneys was calculated as follows: (organ weight)/(body weight) (g/100 g)^29^.

ALT, AST and ALP activities, along with the levels of ALB, BUN and CRE, were measured using an automatic biochemical analyser (ACE, USA).

The pancreas, liver and kidneys were removed rapidly, weighed, and homogenized (1:9, w/v) in phosphate-buffered solution (PBS, 0.2 mol/L, pH 7.4). After centrifugation at 4 °C for 20 min (5000 rpm), the supernatants were collected and assayed for the hepatic/nephritic activities of GSH-Px, SOD, and CAT and MDA levels according to the commercial kit instructions^7^.

Fresh pancreas, liver and kidney tissue masses were fixed in 4% formaldehyde solution (pH 7.4) for more than 24 h, embedded in paraffin and then cut into slices using a microtome. The slices were stained with HE and photographed using an optical microscope (400 × magnification)^7^.

### Acute toxicity

Acute toxicity was determined by the method of Chao *et al*.^52^. Briefly, fifteen Kunming strain mice (4 weeks old, weight: 20 ± 2 g) were randomly divided into one control group, which received the normal saline solution (0.9%), and two dosage groups, which received isometric HIPS1 or HIPS2 solutions at the final concentration of 2000 mg/kg by gavage. The mice were observed continuously for gross behavioural changes, toxic symptoms and mortality during the 14-day feeding period.

### Statistical analysis

Data were statistically analysed using two-way ANOVA and the T-test (SAS, USA). Data were expressed as the means ± SD (standard deviation). Differences were considered statistically significant if *P* < *0.05*.

## Conclusions

The present study demonstrated that HIPS1 and HIPS2 from *H. erinaceus* SG-02 exerted protective effects on the pancreas, liver and kidney in STZ-induced diabetic mice based on the results of biochemical and histopathological analyses. Especially, the HIPS1 was much more efficient in preventing the diabetes-induced organ damages owing to the functional groups (-NH_2_, -COOH and S=O). These results may provide a mechanistic basis for the use of *H. erinaceus* SG-02 as potentially natural and functional foods and drugs for the prevention and alleviation of diabetes and its complications.
